# Suppression of erythroid development *in vitro* by *Plasmodium vivax*

**DOI:** 10.1186/1475-2875-11-173

**Published:** 2012-05-24

**Authors:** Tasanee Panichakul, Witchuda Payuhakrit, Panyu Panburana, Chokdee Wongborisuth, Suradej Hongeng, Rachanee Udomsangpetch

**Affiliations:** 1 Faculty of Science and Technology, Suan Dusit Rajabhat University, 204/3 Sirindhorn Rd., Bangplat, Bangkok, 10700, Thailand; 2 Department of Pathobiology, Faculty of Science, Mahidol University, 272 Rama VI Road, Ratchathewi District, Bangkok, 10400, Thailand; 3 Department of Obstetrics and Gynaecology, Faculty of Medicine, Ramathibodi Hospital, Mahidol University, 272 Rama VI Road, Ratchathewi District, Bangkok, 10400, Thailand; 4 Research Center, Faculty of Medicine, Ramathibodi Hospital, Mahidol University, 272 Rama VI Road, Ratchathewi District, Bangkok, 10400, Thailand; 5 Department of Pediatric, Faculty of Medicine, Ramathibodi Hospital, Mahidol University, 272 Rama VI Road, Ratchathewi District, Bangkok, 10400, Thailand; 6 Center for Emerging and Neglected Infectious Diseases, Mahidol University, 999 999 Phuttamonthon 4 Road, Salaya, Nakhon Pathom, 73170, Thailand

**Keywords:** *Plasmodium vivax*, Erythropoiesis, Haematopoietic stem cells, Anaemia

## Abstract

**Background:**

Severe anaemia due to dyserythropoiesis has been documented in patients infected with *Plasmodium vivax,* however the mechanism responsible for anaemia in vivax malaria is poorly understood. In order to better understand the role of *P. vivax* infection in anaemia the inhibition of erythropoiesis using haematopoietic stem cells was investigated.

**Methods:**

Haematopoietic stem cells/CD34^+^ cells, isolated from normal human cord blood were used to generate growing erythroid cells. Exposure of CD34^+^ cells and growing erythroid cells to *P. vivax* parasites either from intact or lysed infected erythrocytes (IE) was examined for the effect on inhibition of cell development compared with untreated controls.

**Results:**

Both lysed and intact infected erythrocytes significantly inhibited erythroid growth. The reduction of erythroid growth did not differ significantly between exposure to intact and lysed IE and the mean growth relative to unexposed controls was 59.4 ± 5.2 for lysed IE and 57 ± 8.5% for intact IE. Interestingly, CD34^+^ cells/erythroid progenitor cells were susceptible to the inhibitory effect of *P. vivax* on cell expansion. Exposure to *P. vivax* also inhibited erythroid development, as determined by the reduced expression of glycophorin A (28.1%) and CD 71 (43.9%). Moreover, vivax parasites perturbed the division of erythroid cells, as measured by the Cytokinesis Block Proliferation Index, which was reduced to 1.35 ± 0.05 (*P*-value < 0.01) from a value of 2.08 ± 0.07 in controls. Neither TNF-a nor IFN-g was detected in the culture medium of erythroid cells treated with *P. vivax,* indicating that impaired erythropoiesis was independent of these cytokines.

**Conclusions:**

This study shows for the first time that *P. vivax* parasites inhibit erythroid development leading to ineffective erythropoiesis and highlights the potential of *P. vivax* to cause severe anaemia.

## Background

Anaemia has frequently been associated with severe malaria and is believed to contribute to the morbidity and mortality of this disease. Most published reports on malaria-associated anaemia focus on *Plasmodium falciparum* with *Plasmodium vivax* being less well studied [1]. However, growing evidence from several geographic regions has demonstrated that *P. vivax* malaria is associated with a higher frequency and more severe anaemia [2-12]. Several cases of patients infected with *P. vivax* that resulted in severe disease and death were found to have syndromes resembling those commonly observed with falciparum malaria [8,13,14]. Although the underlying causes of severe malarial anaemia are multifactorial, major causes are the destruction of parasitized erythrocytes and ineffective erythropoiesis or dyserythropoiesis. In vivax malaria patients with anaemia red cells in blood film are usually normochromic and normocytic with the absolute reticulocyte count not elevated [15]. However, changes in haemoglobin concentrations are associated with continuing parasitaemia during infection of *P. vivax* in patients with anaemia [6]. In addition, *P. vivax* has been observed in the bone marrow of patients who exhibited dyserythropoiesis [15] and one case of *P. vivax* malaria in Southeast Asia displayed pancytopaenia in blood and bone marrow [16]. Investigation by light and electron microscopy of marrow aspirates from four Thai patients with *P. vivax* malaria and anaemic symptoms revealed morphological evidence of dyserythropoiesis and the presence of erythroblasts at various stages of degradation within the cytoplasm of macrophages [17]. It seems likely that vivax malaria infection was associated with an activation of the pro-inflammatory response and cytokine imbalance [18] and experimental findings in mice are consistent with a role for TNF-a in the dyserythropoietic changes in malaria [19]. Other mechanisms have also been suggested, including alterations in IFN-g, IL-12, IL-6, IL-1, reactive oxygen species, nitric oxide, macrophage dysfunction, or a direct effect of parasites (or parasite products) on the bone marrow [15,20-22]. However, the mechanism responsible for anaemia in vivax malaria remains poorly understood. Here, haematopoietic stem cells (HSCs)/CD34^+^ from normal human cord blood were used to generate growing erythroid cells (gEC) to investigate the effect of *P. vivax* infection on erythropoiesis. Enhancing the understanding of the pathogenesis of anaemia caused by malaria is a prerequisite for developing effective prevention and treatment strategies.

## Methods

### Collection and separation of *Plasmodium vivax* parasites

*Plasmodium vivax* was obtained from patients attending the malaria clinic in Mae Sot, Tak Province, Thailand. Patient blood with 0.1-0.3% parasitaemia, as determined by examining thick and thin blood smears, was collected. The ethical and methodological aspects of this study for parasite collection (MU-IRB 2010/344.1612) have been approved by the Mahidol University Institutional Review Board, Mahidol University, Bangkok, Thailand. Infected erythrocytes (IE) were separated from patient blood using a 60% Percoll solution as previously described [23]. Briefly, whole blood with vivax parasites was collected and then filtrated using a Plasmodipur filter (Euro-Diagnostic B.V., Netherlands) to remove white blood cells. To obtain asexual parasites, packed, infected RBCs from 20 ml of patient blood was diluted 1:2 with RPMI1640 (Invitrogen®, CA, USA), layered on 60% Percoll and centrifuged at 1,200 g for 20 mins at 20°C. The purity of IE after isolation was 95% and the pure fraction of isolated IE contained 80% schizontes and 20% of other stages. The isolated IEs were used either intact or as lysed cells prepared by freezing and thawing.

### Isolation of cord blood CD 34^+^ cells and culture conditions

Umbilical cord blood from normal full-term deliveries in Ramathibodi Hospital, Bangkok, Thailand was collected into cord blood bags containing anticoagulant solution (CPDA-1 solution) (Kawasumi Laboratories, Thailand). Cord blood collection (ID 04-45-16) was approved by the Ethical Committee of Research on Human Beings of the Ramathibodi Hospital, Faculty of Medicine, Mahidol University. Haematopoietic stem cells/CD34^+^ cells were isolated from cord blood mononuclear cells (MNC) using a CD 34 isolation kit with magnetic microbead selection with Mini-MACS columns (Miltenyi Biotech, Geramany) as described by Panichakul *et al*[23]. The purity of CD34^+^ cells after isolation was 97% as judged by flow cytometry analysis.

HSCs/CD34^+^ cells at a density of 1 × 10^5^ cell/well in 24-well tissue culture plates (Corning Incorporated Costar®, NY, USA) were cultured in 0.5 ml of complete medium containing StemlineII medium (Sigma-Aldrich Corporation, Missouri, USA) supplemented with cytokines [23]. IE at the indicated concentration were added to cell cultures on days 1, 5, 8 and 11 and cultured for three additional days. Intact and lysed IE from the same patients were utilized in this study. Recombinant human tumour necrosis factor-alpha (TNF-a), human interferon gamma (IFN-g) (Prospec-Tany TechnoGene Ltd., Rehovot, Israel), and uninfected erythrocytes (UE) from normal donor blood were included in this study. All cultures were incubated at 37°C in 5% CO_2_ and viable cells were determined by trypan blue dye exclusion.

### Detection of surface markers of erythroid cells

Cell surface markers were detected using immunofluorescence with mouse antibodies to human CD34 (Miltenyi Biotech), CD71, CD45 (eBioscience, Inc. CA, USA) and glycophorin A (Serotec Inc., NC, USA) to confirm cell types of HSC and derived erythroid cells. Cells (5–10 × 10^4^ cells in 100 μl of medium) were stained with 5 μl of each antibody for 30 min at 4°C. After washing twice with phosphate-buffer saline, stained cells were fixed with 1% paraformaldehyde. Cell death was also determined by staining with 20 μg/ml of propidium iodide (BenderMedSystems®, GmSH). All cell markers were analysed by flow cytometry using an Epics XL-MCL analyser (Beckman Coulter, Inc. CA, USA).

### Determination of inflammatory cytokines

The presence of inflammatory cytokines TNF-a and IFN-g in culture medium of gEC was determined using the Bio-Plex Pro™ Magnetic Cytokine assay (Bio-Rad Laboratories, Inc. CA, USA). Briefly, 50 μl of beads coated with different anti-cytokine antibodies were added to a pre-wet filter plate and after washing 50 μl of culture medium or cytokine standards were added to duplicate wells and incubated for 30 min at room temperature in the dark. The plate was washed three times with washing buffer and 50 μl of streptavidin-PE was added and the reaction was incubated for 10 min. Following three washes, 125 μl of assay buffer was added and the plate was analysed immediately using an array reader (Bio-Plex Manager 5.0 Software, Bio-Rad Laboratories).

### Cell division assay

Five-day old cells were exposed to lysed IE for three days followed by incubation with 3 μg/ml cytochalasin B (Sigma-Aldrich Corporation) for 24 h as previously described [24]. Cells (5–10 x 10^4^ in 100 μl of medium) were spun onto a slide using cytospin (Cytospin 3, Thermo Shandon, UK) at 800 rpm for 10 min then fixed with 95% ethanol for 10 min. Thereafter, cells were stained for 10 min with Giemsa, then washed and dried. Stained cells were examined under a light microscope (Olympus BX50, Japan) and the proportion of mono-, bi-, tri- and tetranucleated cells was evaluated in samples of 2,000 cells. The Cytokinesis Block Proliferation Index (CBPI) was calculated using the formula CBPI = X(1 N) + Y(2 N) + Z(3 N) / X + Y + Z; where X, Y and Z are the number of cells with one, two and three nuclei (N), respectively [25].

### Statistical evaluation

Data were analysed using the SPSS program (version 11.0). The unpaired Mann–Whitney - Wilcoxon test was used to compare means between independent groups as appropriate and for statistical evaluation of CBPI. Results are reported as statistically significant if the *P-*value was less than 0.01.

## Results

### Inhibition of cell growth by *Plasmodium vivax*

**Figure 1:**
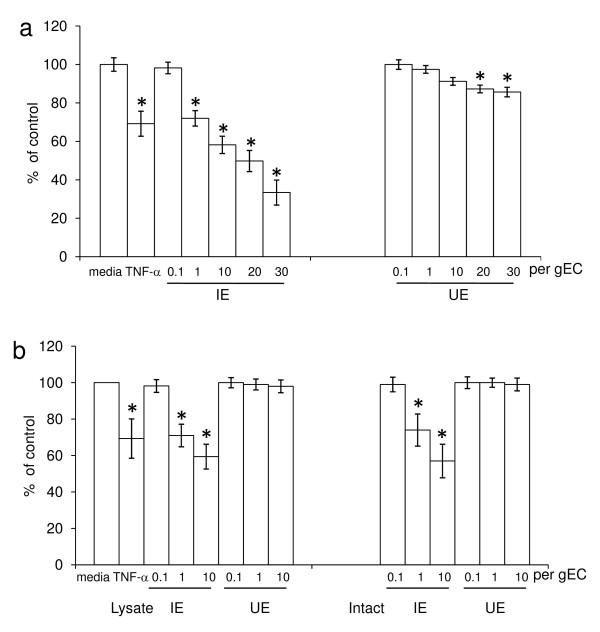
**Effect of*P. vivax*on cell growth*.*(a)** Lysates of infected erythrocytes (IE) or uninfected erythrocytes (UE) and **(b)** lysed IEs or UEs and intact IEs or UEs were added in varying ratios of IE numbers to one growing erythroid cells (gEC) on day 5. Erythroid growth was evaluated on day 8. The mean of erythroid growth (shown in bar graphs) was compared with a control containing medium without IE or UE lysate. 25 ng/ml of TNF-a was used as a positive control. The error bars show the S.D. of 5 independent experiments, *P*-value < 0.01 compared with medium control.

Growing erythroid cells (gEC) derived from human cord blood HSCs/CD34^+^ cells were used as an *in vitro* model for studying changes in erythropoiesis caused by *P. vivax* infection. Infected erythrocytes (IE) were isolated from the blood of malaria patients and the effect of the presence of IE on erythroid growth was determined as shown in Figure 1a. The effect of lysates from IE was compared with those from uninfected erythrocytes (UE) on gEC cultures by treating cells on day-5 with five ratios of IE/UE: gEC (0.1:1, 1:1, 10:1, 20:1 and 30:1). After culturing gEC for another three days, parasite-induced inhibition of erythroid growth was monitored and ratios as low as 1 lysed IE to 1 gEC were found to cause a dose-dependent decrease in growth (Figure 1a). The effect of lysed and intact IE on erythroid growth was evaluated and is shown in Figure 1b. Lysed and intact IE or UE were added on day 5 of gEC cultures and their effects on day 8 were compared. Both lysed and intact IE at a IE : gEC ratio of 10:1 significantly inhibited the erythroid growth however addition of UE did not alter growth. The reduction of erythroid growth did not differ significantly between intact and lysed IE with the mean growth relative to controls without lysed or intact IE was 59.4 ± 5.2 for lysed IE and 57 ± 8.5% for intact IE. These results demonstrated that there was no metabolic effect of viable infected cells on erythroid cell growth. Similar results were obtained with infected erythrocytes from five different parasite isolates. Overall these results demonstrate that lysed IE with low numbers of *P. vivax*-infected cells are sufficient to inhibit the growth of erythroid cells.

### Inhibition of erythroid development

**Figure 2:**
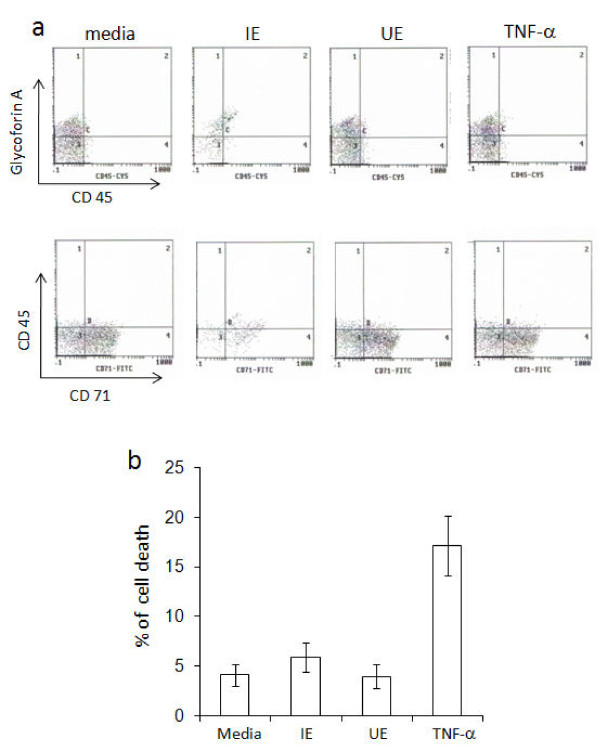
**Determination of erythroid cell markers and death.** gECs, five-days old were cultured with IE or UE lysate in the ratio 1:10. Cell markers were determined after culturing for another three days by flow cytometry. **(a)** Dot-scatter graphs of erythroid cell markers, using fluorescence-labelled anti-glycophorin A, CD 45 and CD 71 antibodies, and **(b)** cell death, using propidium iodide (PI) staining. The error bars of histograms show the S.D. of three independent experiments.

The inhibition of erythroid expansion and differentiation by *P. vivax* was evaluated in the presence of IE and UE. Lysed IE or UE were mixed with isolated CD34^+^ cells/erythroid progenitor cells on day 1, 5, 8 and 11 after induction of erythroid development. After three days, lysed IE inhibited erythroid expansion, compared with the controls (Table 1). The effect was greatest when a ratio of 10 IE to one erythroid precursor was used on day-1 resulting in reduction of erythroid growth to 43 ± 5.5% compared with controls without IE. The degree of inhibition of erythroid expansion by IE on days 5, 8 and 11 was gradually decreased but still significant (*P*-value < 0.01) indicating that CD34^+^/erythroid progenitor cells were susceptible to the inhibitory effect of *P. vivax* on cell expansion. The susceptibility to the inhibitory effect of *P. vivax* was decreased when cells were mature as shown in Additional file 1. In addition, a reduction in erythroid cell development was supported by the observed decrease in the expression of erythroblast markers, 28.1% of glycophorin A and 43.9% of CD 71 after three days of culture with IE lysates compared to controls without IE which expressed 50% of glycophorin A and 72.1% of CD 71 (Figure 2a). Cell death was also determined and found to occur at similar levels in cultures with IE lysate (5.9 ± 1.2%) or media alone (4.1 ± 1%), after three days of culture (Figure 2b). This indicates that the reduction of erythroid cells when exposed to IE lysates was not from death activation from parasites but rather that parasites suppress erythropoiesis through an inhibitory effect on expansion of CD34^+^/erythroid progenitor cells.

**Table 1:** **Inhibition of erythroid expansion by lysate of infected erythrocytes to CD34**^**+**^**cells /erythroid progenitor cells**

Day-old of cell cultures	% of control (mean ± S.D.)				
	IE	IE	UE	UE	TNF-a
	1	10	1	10	25 ng/ml
1	67 ± 9.5*	43 ± 5.5*	100 ± 4.5	98 ± 5.5	54 ± 12*
5	72 ± 6.5*	58 ± 4.5*	99 ± 4.0	98 ± 4.8	69 ± 8*
8	88 ± 4.5	65 ± 7.5*	99 ± 2.0	99 ± 5.5	74 ± 7*
11	98 ± 8.5	87 ± 7.0*	100 ± 4.5	100 ± 5.0	78 ± 6*

* *P*-value < 0.01 compared with control wells.

Inhibition is presented as percentage of expansion of the control after three day-culture without added medium, cells, or TNF-a. Lysates of infected erythrocyte (IE) and uninfected erythrocyte (UE) were added at ratios of 1 or 10 IE / UE per 1 CD34^+^ cells / erythroid progenitor cells. The mean and S.D. were calculated from five independent experiments.

### Suppression of cell division by *Plasmodium vivax*

**Figure 3:**
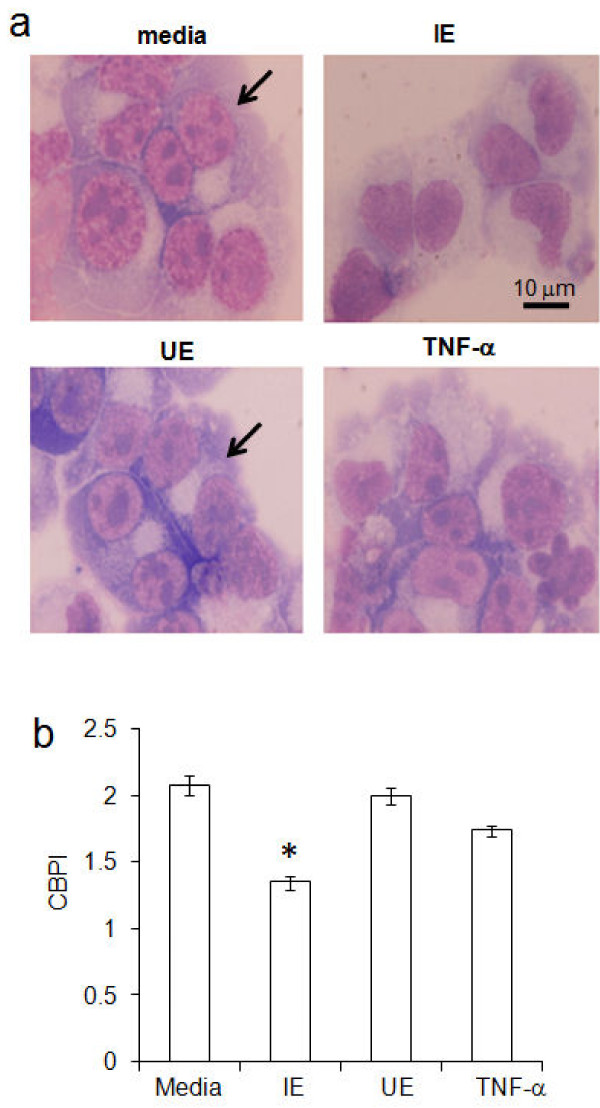
**Reduction of cell division by* P. vivax*.** Five-day old growing erythroid cells (gECs), 10^5^ cells, in cultures with added media, lysates of 10^6^ infected cells (IE) or uninfected cells (UE) and TNF-a were incubated for 72 h. **(a)** Cells stained with Giemsa, bi-nucleuses in gECs are indicated with arrows, magnification X 1,000. **(b)** CBPI (cytokinesis block proliferation index) of gEC cultures after added cytochalasin B. The error bars represent the S.D. for five independent experiments, *P-value < 0.01.

Cytokinesis Block Proliferation Index (CBPI) is a useful parameter for assessing the inhibition of cell division in cultures. To examine the inhibition of erythroid division by *P. vivax*, lysed IE were added to five-day old gEC cultures and after three days cell division was blocked by cytochalasin B. In controls, treated with media or UE lysates, large numbers of bi-nucleated cells were observed with few tri- and tetra-nucleuated gEC. In contrast, cultures exposed to IE lysates contained high numbers of mono-nucleus gEC (Figure 3a). Cultures undergoing parasite-suppression due to exposure with IE lysates displayed a significant reduction (*P*-value < 0.01) in the CBPI (1.35 ± 0.05; mean ± S.D.) compared with gEC in media control (2.08 ± 0.07) as shown in Figure 3b. The CBPI of cultures treated with UE lysate and TNF-a were 2.0 ± 0.06 and 1.74 ± 0.04, respectively. These results suggest that vivax parasites inhibited expansion of erythroid progenitor cells by blocking cellular division.

**Figure 4:**
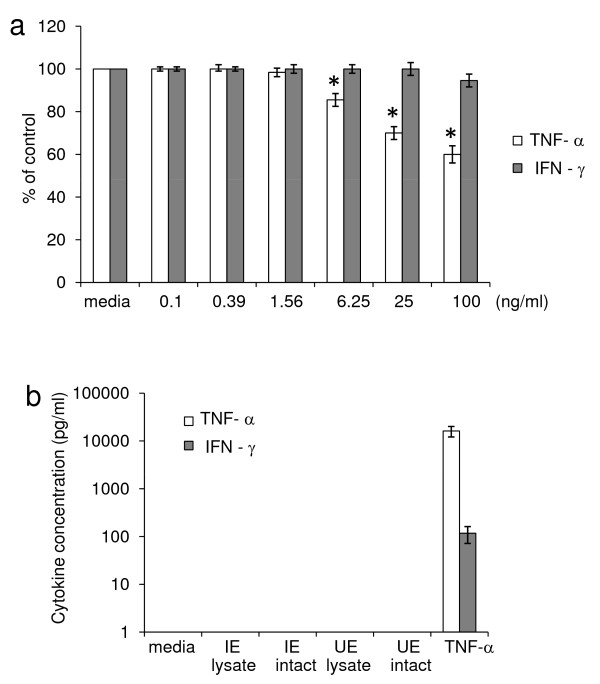
**Effect of inflammatory cytokines on erythroid cell growth.(a)** Growing erythroid cells (gECs) 5-days old were treated with various concentrations of TNF-a (white bars) or IFN-g (grey bars) and cultured for an additional three days. * *P* < 0.01 compared with medium control. **(b)** TNF-a (white bars) and IFN-g (grey bars) in erythroid culture activated by intact IEs or UEs; lysates of IEs or UEs; and 25 ng/ml of TNF-a as a positive control were determined using the Bio-Plex Pro™ Magnetic Cytokine assay. The error bars show the S.D. from three independent experiments.

In patients with vivax malaria, TNF-a and IFN-g have been associated with malaria anaemia and may be involved in promoting inadequate erythropoiesis. In this model system, gEC cells were treated with recombinant human TNF-a or IFN-g in various concentrations and the erythroid cell growth was inhibited at TNF-a and IFN-g concentrations 6.25 and 100 ng/ml, respectively (Figure 4a). However, after adding lysed or intact IE to the gEC cultures on day 5 endogenous TNF-a was undetectable after three days of parasite activation (Figure 4b), even though the lower limit of detection in this assay was 5 pg/ml. Another cytokine, IFN-g, was also undetectable in medium cultured with intact or lysed IE. However, IFN-g could be detected in cultures treated with 25 ng/ml of TNF-a, as shown in Figure 4b. The lower limit of IFN-g detection in this assay was 26 pg/ml. Therefore, in this model, parasites inhibited erythroid development in a way that was independent of TNF-a and IFN-g.

## Discussion

Numerous reports have shown that *P. vivax* can be associated with severe anaemia [2-12]. Haematologic profiles of pancytopaenia in blood and bone marrow [16] and dyserythropoiesis in bone marrow [15] have been reported in vivax malaria patients. However, the cause of reduction in blood cell production in the bone marrow of patients with vivax malaria is not completely understood. Anaemia in malaria is caused by excessive removal of non-parasitized erythrocytes, the immune destruction of parasite-infected red cells, as well as by the impaired compensation due to bone marrow dysfunction [1,15,17,26-28]. *Plasmodium vivax* requires reticulocytes for expansion of the blood stages [29] and parasitaemia is generally low, therefore it is unlikely to be the primary cause of anaemia [6]. This suggests that in addition to the simple destruction of infected red cells another mechanism is involved in anaemia in vivax malaria.

Recently, the production of erythrocytes from the *in vitro* cultures of haematopoietic stem cells was achieved, as previously described [23] and this model is now being applied to dissect the complexity of anaemia in malaria. The results presented in this study have revealed for the first time that *P. vivax* can directly inhibited erythropoiesis, as shown by the reduction of erythroid growth in the presence of either lysed or intact IE. Erythroid progenitor cells were susceptible to the inhibitory effect of *P. vivax* on cell expansion and this result is consistent with the previous report that young stages of erythroid cells were more susceptible to *P. vivax* infection [23]. The suppression of erythropoiesis in malarial anaemia is not unique to *P. vivax* and has also been observed in infections from other *Plasmodium* species*.* In the complicated *Plasmodium falciparum* infection, erythroid suppression is indicated by a decrease in the number of erythroid precursors as well as colony-forming units-erythroid (CFU-E) and burst-forming units-erythroid (BFU-E) in the bone marrow cultures [30]. *Plasmodium chabaudi* can directly suppress the proliferation, differentiation and maturation of erythroid progenitor cells and causes inadequate reticulocytosis in mice [31]. However, deficient erythropoietin production does not appear to be the cause of inadequate erythropoiesis in malaria [32].

Decreased responsiveness of erythroid progenitor cells to erythropoietin as well as impaired erythropoietin production mediated by inflammatory cytokines has been reported to be involved in anaemia during inflammation [33]. Consistent with this observation, TNF-a was reported to partly inhibit proliferation of erythroid progenitor cells in bone marrow cultures [34]. Erythroid progenitor cells produced in this model were also susceptible to inhibition by exogenous TNF-a as shown in Figure 4a. However, endogenous TNF-a and IFN-g in erythroid cultures exposed to lysates or intact *P. vivax* was undetectable (Figure 4b). This suggests that *P. vivax* can also inhibit erythropoiesis independently of TNF-a and IFN-g. Inhibition of erythroid development that is independent of TNF-a and IFN-g has also been observed by exposure with *P. falciparum* haemozoin [35,36]. However, other inflammatory cytokines may be involved and high levels of IL-10 were found to correlate positively with inhibition of proliferative peripheral blood mononuclear cells in the presence of *P. falciparum* haemozoin [37]. In this study, IL-10 was also detectable in supernatants from gECs in the presence of IEs and the role of this cytokine in the inhibition of erythropoiesis is currently being investigated. Interestingly, *P. vivax* inhibited not only growth but also the differentiation of erythroid progenitor cells as shown by the reduction of glycophorin^+^ and CD 71^+^ cells and this is similar to the inhibitory effect of *P. falciparum* haemozoin on erythroid cell development [35,38]. Moreover, vivax parasites were able to perturb the cell division but did not induce the cell death of erythroid progenitor cells. Defects in the cell cycle without apoptosis has also been observed with the inhibitory effect of *P. falciparum* haemozoin on erythroid cell growth [38]. It was found that *falciparum* haemozoin-treated erythroid cells enhanced the expression of the transcription factor p53 and cdk-inhibitor p21 in addition the retinoblastoma protein, a central regulator of G- to S-phase transition was hypophosphorylated, while GATA-1, the master transcription factor in erythropoiesis was reduced [38]. Therefore the molecular mechanisms underlying the suppression of erythropoiesis by *P. vivax* or its products warrants further investigation. The findings of this study are consistent with the hypothesis that vivax parasites can suppress erythropoiesis. These results provide a better understanding of the role of chronic and persistent *P. vivax* infection as a cause of anaemia. Prolonged exposure to vivax parasites can suppress erythropoiesis as well as inhibit reticulocyte production, which could prevent the restoration of the erythrocyte population in chronic parasitaemic *P. vivax* infection. Many cases of patients with severe anaemia have been reported in vivax endemic areas in Thailand, Indonesian Papua, Korea, Pakistan, Venezuela, and Colombia [3,5,7,9,10,14]. These patients are often infected or re-infected with the vivax parasites and parasites have the potential to inhibit erythroid development leading to ineffective erythropoiesis causing severe anaemia.

## Conclusions

This finding suggests that suppression of erythropoiesis by *P. vivax* infection is potentially much more dangerous than it is commonly believed and defective erythropoiesis should be taken into consideration in the development of therapeutic strategies to treat severe malarial anaemia.

## Abbreviations

HSC: Haematopoietic stem cell; MNC: Mononuclear cell; IE: Infected erythrocyte; UE: Uninfected erythrocyte; gEC: Growing erythroid cell; TNF-a: Tumour necrosis factor-alpha; IFN-g: Interferon-gamma; CBPI: Cytokinesis block proliferation index; CFU-E: Colony-forming units-erythroid; BFU-E: Burst-forming units-erythroid.

## Competing interests

The authors declare that they have no competing interests.

## Authors’ contributions

TP designed the study, collected vivax parasites from patients, performed experiment and statistical analysis, and wrote the manuscript. WP isolated human CD34^+^ cells and cultured cells. PP collected human cord blood from normal full-term deliveries. CW performed analysis of cell markers and cytokine assay. SH and RU contributed substantially to the design of the study and critically revised the manuscript. All authors read and approved the final manuscript.

## Supplementary Material

Additional file 1: **Erythroid cell development.** Giemsa staining of cells from one to 11 day-old cultures showing CD34^+^ cells on day 1, and morphological characteristic of erythroid cells with haemoglobin and chromatin condensation for an orthochromic normoblasts (arrow) on day 11. Magnification X 1,000. Click here for file
