# Supplementary material for: Suppression of erythroid development in vitro by Plasmodium vivax
Source: Malar J. 2012 May 24;11:173. doi: 10.1186/1475-2875-11-173 (PMC3407695; doi:10.1186/1475-2875-11-173)
Supplement: Additional file 1: — Erythroid cell development. Giemsa staining of cells from one to 11 day-old cultures showing CD34+ cells on day 1, and morphological characteristic of erythroid cells with haemoglobin and chromatin condensation for an orthochromic normoblasts (arrow) on day 11. Magnification X 1,000. [file 1475-2875-11-173-S1.pdf]

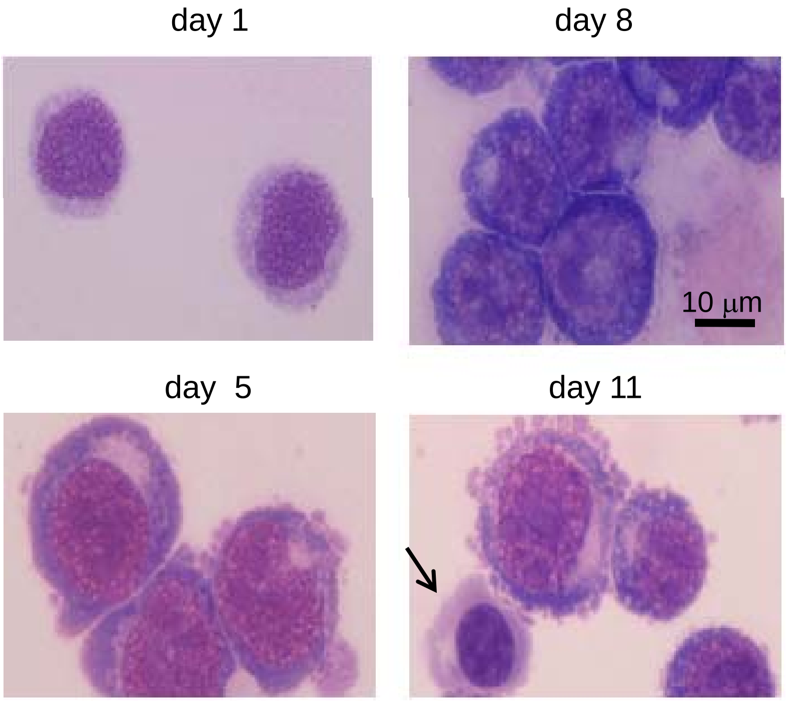

Additional file 1 Erythroid cell development. Giemsa staining of cells from 1-11 day-old cultures showing $CD34^+$ cells on day 1, and morphological characteristic of erythroid cells with hemoglobin and chromatin condensation for an orthochromic normoblasts (arrow) on day 11. Magnification X 1,000.
